# The obese transplant organ recipient: experimental and clinical evidence for tailored immunosuppression

**DOI:** 10.3389/ti.2026.16006

**Published:** 2026-05-11

**Authors:** A. Della Penna, S. Beer-Hammer, S. Nadalin, I. Capobianco, P. Felgendreff, M. Schmelzle, M. Quante

**Affiliations:** 1 Department of General, Visceral and Transplantation Surgery, University Hospital Tübingen, Tübingen, Germany; 2 Department of Pharmacology, Experimental Therapy and Toxicology, Institute of Experimental and Clinical Pharmacology and Pharmacogenomics, University Hospital Tübingen, Tübingen, Germany; 3 Hannover Medical School, Department of General, Visceral and Transplant Surgery, Hannover, Germany

**Keywords:** immunosuppression, obesity, pharmacokinetic, precision medicine, solid organ transplant (SOT)

## Abstract

Obesity has become a major determinant of outcomes across solid organ transplantation. Beyond its well-recognized metabolic and cardiovascular burden, obesity profoundly affects both immune regulation and the pharmacology of immunosuppressive therapy. Experimental evidence has established adipose tissue as an active immune organ that promotes low-grade inflammation through leptin, TNF-α, and IL-6, thereby altering alloimmune responses and impairing graft tolerance. Clinically, obesity is associated with increased surgical complications, delayed graft function, and reduced survival after kidney, liver, and thoracic organ transplantation. In parallel, obesity modifies drug disposition at every pharmacokinetic step, expanding the distribution volume for lipophilic agents such as calcineurin and mTOR inhibitors, altering CYP3A metabolism, and increasing interindividual variability in exposure. Consequently, both underexposure and toxicity remain frequent, underscoring the need for individualized therapeutic strategies. Current evidence supports the integration of therapeutic drug monitoring, pharmacogenomics, and biomarker-based approaches to refine immunosuppression intensity. This review summarizes experimental and clinical data linking obesity-induced inflammation with altered immunosuppressive pharmacology and proposes a framework for precision immunosuppression that balances efficacy, nephroprotection, and metabolic safety. Tailoring therapy to the specific immunometabolic profile of obese recipients may thus transform a major clinical challenge into an opportunity for precision transplant medicine.

## Introduction

The prevalence of obesity among solid organ transplant recipients has risen steadily over recent decades in parallel to global population trends [1, 2]. Registry analyses indicate higher mean BMI at the time of listing and transplantation nowadays when compared with historical cohorts [3–5]. This epidemiological shift has major clinical implications, as obesity is associated with increased perioperative risk, altered pharmacokinetics of immunosuppressive agents, and long-term metabolic complications that can compromise graft function [6–8]. Obesity has therefore transitioned from a secondary comorbidity to a primary determinant of transplant outcomes, underscoring the need for risk-adapted recipient selection, perioperative management, and individualized immunosuppressive strategies [9, 10]. This narrative review aims to provide an integrated overview of obesity-related immunological and pharmacokinetic alterations in transplantation, and to define a framework for individualized immunosuppressive strategies in obese recipients.

## Clinical graft outcome in obese recipients

### Kidney

Kidney transplantation is consistently associated with poorer outcomes in obese recipients. Large registry analyses demonstrate that severe obesity independently impairs graft and patient survival [4, 11]. Meta-analyses confirm that obesity increases the incidence of delayed graft function, surgical complications, and even mortality [7, 12]. Recent multicenter data suggest that these risks persist despite advances in immunosuppressive protocols and perioperative care [13, 14]. Histopathological evidence links obesity with renal microvascular injury and chronic inflammatory graft infiltration, as described in obesity-related glomerulopathy with glomerulomegaly, mesangial expansion, focal segmental glomerulosclerosis, interstitial fibrosis and immune cell infiltration [15, 16].

### Liver

Obesity may have negative implications on both candidacy and post-transplant outcomes in liver transplantation. Here, obese candidates often face higher surgical risk and comorbidity burdens, consecutively impacting listing eligibility [8, 17]. After transplantation, obesity is associated with increased rates of wound complications, prolonged hospital stays, and most importantly diminished survival [8, 18, 19]. Here, meta-analytic data confirm higher perioperative morbidity and long-term mortality in obese liver recipients [8, 19]. Of note, emerging evidence reveals sex-specific patterns in obesity-related liver transplant outcomes. For instance, female recipients with elevated BMI undergoing DCD liver transplantation are carrying a higher risk of early graft rejection [20], while among NASH-related hepatocellular carcinoma cases, women had significantly lower post-transplant mortality than men [21]. Hormonal milieu and metabolic derangements—especially in post-menopausal women—add further complexity to this dynamic processes [22].

### Thoracic organs

Beyond liver and kidney transplantation, obesity is also impacting outcomes in the clinical context of thoracic organ transplantation. For heart transplantation, excess body weight has been associated with increased perioperative risk and inferior long-term survival, particularly due to the higher prevalence of cardiovascular and metabolic comorbidities in obese recipients [23]. Moreover, in a large cohort study, obese heart transplant recipients demonstrated a significantly higher risk of death, primary graft dysfunction, and any treated rejection [24].

In lung transplantation, accumulating evidence indicates that obesity is an independent predictor of adverse outcomes. Gries et al. demonstrated that obese recipients with idiopathic pulmonary fibrosis have a significantly increased 90-day mortality risk following bilateral lung transplantation [25]. Along the same lines, BMI has been shown to be a strong predictor of early mortality within the first 90 days post lung-transplant [26], while long-term follow-up revealed lower overall survival among overweight and obese recipients compared to their normal-weight counterparts [27].

Taken together, these observations highlight that the deleterious effects of obesity extend beyond abdominal organ transplantation and are particularly relevant in thoracic organs, where perioperative complications, impaired wound healing, and cardiopulmonary stress further aggravate the pre-existing risk profile of obese transplant recipients [2].

### Perioperative considerations

Beyond organ-specific outcomes, obesity is also linked to relevant surgical and perioperative challenges that may influence early graft outcomes and, consequently, immunosuppressive management. Increased adipose tissue and altered anatomy are associated with increasing operative complexity, including prolonged procedure and ischemia times, as well as technical difficulties during vascular anastomosis [28]. From an anesthesiological perspective, obese recipients are at higher risk of perioperative respiratory complications and hemodynamic instability, further contributing to early postoperative vulnerability [29].

Wound-related complications still represent one of the most consistent findings across organ types, with obesity being strongly associated with higher rates of surgical site infections, wound dehiscence, and prolonged hospitalization [30]. In kidney transplantation, obesity is also an independent risk factor for delayed graft function, likely reflecting a combination of technical factors, ischemia–reperfusion injury, and underlying inflammatory alterations [7].

These perioperative factors have direct implications for early immunosuppressive management. On the one hand, increased rates of wound complications and infection may favor more cautious immunosuppressive exposure in the immediate postoperative phase. On the other hand, the higher risk of delayed graft function and obesity-related immune activation may require adequate immunosuppressive intensity to prevent early rejection. This clinical tension underscores the need for careful balancing of efficacy and safety, further calling for an individualized approach to immunosuppression in obese transplant recipients.

## Obesity promoting inflammation

### Experimental evidence

Seminal studies defined adipose tissue as an active immune organ rather than a passive energy reservoir [31, 32]. Here, tumor necrosis factor-α production from adipose tissue was first identified as a link between adiposity and systemic inflammation [33]. Furthermore, leptin, an adipocyte-derived hormone, was shown to regulate both metabolic and immune processes [34, 35]. Adipose tissue was subsequently recognized as a major source of interleukin-6, while adiponectin—an anti-inflammatory adipokine—was found to be reduced in obesity [36, 37]. Macrophage infiltration into adipose depots is a further key driver of the chronic low-grade inflammatory state characteristic of obesity [38, 39]. Recent reviews reinforce these findings, detailing the regulation of immunometabolism within adipose tissue [40] and highlighting macrophage recruitment dynamics in obesity-related adipose tissue inflammation [41].

### Clinical evidence

**FIGURE 1:**
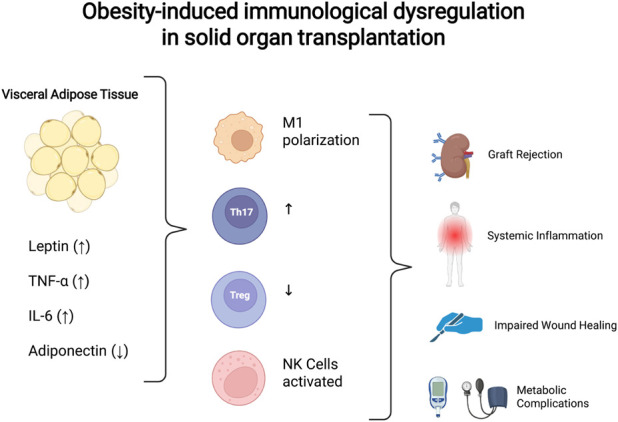
Obesity-associated immunological dysregulation relevant to solid organ transplantation. Schematic representation of the pro-inflammatory milieu induced by visceral adiposity, characterized by increased leptin, TNF-α, and IL-6 levels, reduced adiponectin, and macrophage M1 polarization. Obesity promotes natural killer cell activation, systemic inflammation, impaired wound healing, and metabolic complications. These alterations converge to amplify alloreactivity and increase the risk of graft rejection, thereby linking adipose-driven immune dysregulation with inferior transplant outcomes.

Clinical studies confirm that obesity-induced inflammation is both measurable and clinically significant in the context of solid organ transplantation. Visceral adiposity is associated with increased immune activation and elevated systemic inflammatory markers compared to subcutaneous depots [42, 43]. In transplant recipients, obesity is accompanied by a pro-inflammatory state that directly contributes to poorer graft outcomes [9, 44]. Evidence from clinical kidney and liver transplantation confirms that the systemic inflammation characteristic of obesity is a major determinant of reduced graft survival [44]. These observations establish obesity-related inflammation as a key regulator linking obesity to inferior transplant outcomes [10, 32] (see Figure 1).

## Immunosuppression and obesity

**FIGURE 2:**
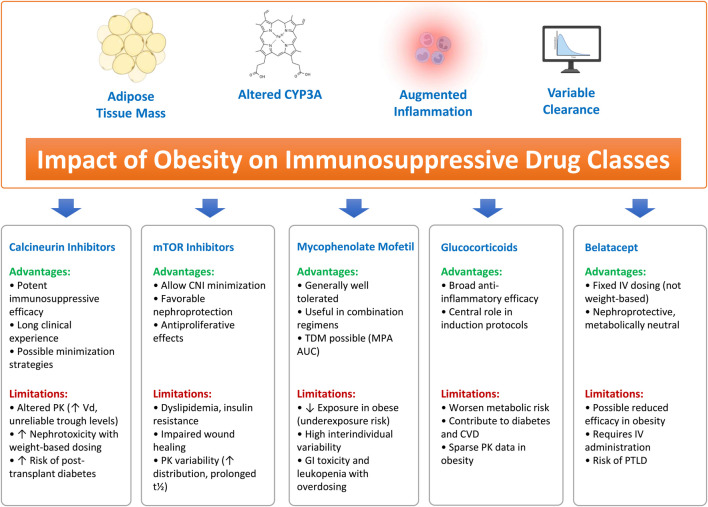
Impact of obesity on pharmacokinetics and clinical performance of major immunosuppressive drug classes. Overview of how increased adipose mass, altered CYP3A activity, and inflammation modulate drug disposition in obese recipients. Calcineurin inhibitors exhibit expanded volume of distribution, unreliable trough levels, and enhanced nephrotoxicity with weight-based dosing. mTOR inhibitors demonstrate variable exposure, prolonged half-life, and increased metabolic toxicity. Mycophenolate mofetil shows reduced MPA exposure in heavier patients, while glucocorticoids exacerbate metabolic risk with limited obesity-specific PK data. Belatacept, administered as fixed dosing, may display altered clearance and reduced efficacy in obesity. Together, these obesity-driven changes underscore the need for individualized dosing and careful therapeutic monitoring.

Obesity has profound impact on the pharmacokinetics and pharmacodynamics of immunosuppressive drugs. Here, alterations may occur at virtually every step of drug disposition: absorption, distribution, metabolism, and clearance [45, 46]. Increased adipose mass and lean body mass expand the volume of distribution for lipophilic agents, while hepatic steatosis and comorbid metabolic syndrome may impair drug metabolism [47]. Moreover, renal hyperfiltration in obesity alters the clearance of renally excreted metabolites [48]. These metabolic-driven changes translate into significant variability in drug exposure, complicating therapeutic drug monitoring and raising the risk of both rejection and toxicity [49, 50] (see Figure 2).

### Calcineurin inhibitors (tacrolimus and cyclosporine)

Calcineurin inhibitors remain the cornerstone of most immunosuppressive regimens. Both tacrolimus and cyclosporine are highly lipophilic, extensively protein-bound, and metabolized by CYP3A enzymes, thus making them highly susceptible to obesity-related alterations [45, 46]. Obesity increases the apparent volume of distribution, while trough concentrations do not reliably reflect systemic exposure [51]. Tacrolimus in particular shows reduced predictability of trough levels in obese recipients, therefore complicating standard monitoring approaches [49, 52]. Cyclosporine clearance and distribution were already shown decades ago to be significantly altered by obesity [53]. Clinically, these pharmacokinetic changes translate into an increased risk of overexposure and nephrotoxicity. When using conventional weight-based dosing, whereas fixed or capped dosing strategies may reduce toxicity, but require close monitoring to avoid underexposure in fast metabolizers [54]. Further data suggest that full-dose CNI regimens in obese recipients are disproportionately nephrotoxic [51]. Consequently, minimization strategies and individualized monitoring are highly recommended [55]. Current practice increasingly favors fixed dosing with careful monitoring, rather than strict weight-based dosing, to avoid systematic overdosing in obese patients [49].

### Mechanistic target of rapamycin (mTOR) inhibitors (sirolimus and everolimus)

Inhibitors of mTOR are also highly lipophilic and demonstrate significantly altered pharmacokinetics in obesity [56, 57]. In detail, sirolimus has a prolonged half-life and greater distribution in obese recipients, increasing the risk of cumulative toxicity [58]. Everolimus, though shorter-acting, has been associated with increased metabolic complications in obese recipients, particularly dyslipidemia and post-transplant diabetes [59, 60]. These metabolic toxicities align with the pro-inflammatory and insulin-resistant milieu of obesity, thus finally compounding the cardiovascular risk [59].

While mTOR inhibitors facilitate CNI minimization, advantageous for nephrotoxicity-prone obese patients, their potential to worsen dyslipidemia and insulin resistance may offset these benefits, particularly in metabolically fragile individuals [55]. Recent registry analyses further support this concept: in an SRTR cohort, regimens combining mTOR inhibitors with tacrolimus were associated with reduced acute rejection rates in obese kidney transplant recipients, suggesting tailored benefits in this subgroup [61]. In contrast, combinations of mTOR inhibitors with mycophenolate mofetil have consistently been associated with inferior efficacy and increased toxicity, and are therefore neither recommended in obese recipients nor in the general transplant population [61]. Clinical application therefore requires judicious patient selection and close metabolic monitoring [62]. Fixed dosing strategies with trough-level adjustment remain standard, but variability in obese recipients suggests that more refined AUC-based or model-informed precision dosing could improve safety [63].

### Mycophenolate mofetil (MMF)

As a hydrophilic prodrug converted to mycophenolic acid (MPA), MMF demonstrates high interindividual variability in exposure [64]. Body weight has been identified as a major determinant of exposure variability: data from the OPTICEPT trial showed that heavier kidney transplant recipients had significantly lower MPA area under the curve (AUC) per mg dose compared with lighter patients, despite identical dosing regimen [65]. A systematic evaluation of clinical practice confirmed that individualized dosing based on therapeutic drug monitoring (TDM) can optimize exposure in patients with high pharmacokinetic variability, including those with obesity [50]. Clinically, suboptimal MMF exposure in obese recipients can contribute to breakthrough rejection, while overdosing increases the risk of leukopenia and gastrointestinal toxicity [66]. Because MMF is typically applied with fixed dosing, obesity poses challenges in predicting systemic exposure. This makes TDM of MMF particularly valuable in obese transplant recipients [67]. Unfortunately, routine AUC monitoring is rarely implemented outside specialized centers, representing an unmet need in current practice. A prospective multicenter study confirmed that individualized AUC-based MMF dosing significantly improves clinical outcomes after renal transplantation [68].

### Glucocorticoids

Steroids still remain a backbone of induction and maintenance therapy, though their utilization has declined due to their well-known metabolic side effects [69, 70]. Obesity modifies glucocorticoid metabolism, leading to altered efficacy and increased risk of complications such as weight gain, diabetes, and cardiovascular disease [71, 72]. Evidence for steroid minimization or withdrawal in obese recipients suggests potential benefits in reducing metabolic complications, but these strategies carry an increased risk of graft rejection [69, 70]. While steroid minimization or withdrawal strategies are often pursued to mitigate the well-documented metabolic adverse effects of glucocorticoids, increasing evidence suggests that obesity and post-transplant weight gain may still occur independently of steroid exposure. For example, in a cohort of kidney transplant recipients managed with steroid avoidance, Elster et al. reported significant weight gain despite the absence of maintenance glucocorticoids [73]. These findings indicate that although glucocorticoids are a major driver of post-transplant metabolic complications, additional mechanisms—including pre-existing obesity, immunosuppressive drug classes such as CNIs or mTOR inhibitors, and lifestyle factors—may substantially contribute to post-transplant adiposity. Pharmacokinetic data on steroids in obese transplant recipients are sparse, thus reflecting a critical gap of knowledge [69]. Unlike for CNIs and mTOR inhibitors, systematic obesity-stratified pharmacological studies of glucocorticoids are virtually absent, leaving dosing largely empirical [74]. Given their profound impact on the individual patients’ metabolic risk, more focused studies are needed to optimize steroid use in obesity [71].

### Belatacept (CTLA4-Ig)

Belatacept, a fusion protein targeting the costimulatory ligands CD80/86, is offering an appealing alternative to CNIs [75]. Unlike small molecules, it is administered at fixed intravenous doses, largely independent of body weight [76]. Importantly, efficacy of belatacept has been demonstrated in kidney transplant recipients, including those with obesity, with a significant lower risk of nephrotoxicity compared to CNIs [77]. However, recent pharmacokinetic data indicate altered clearance in obese patients, thus raising the possibility of under- or overexposure with fixed dosing [78].

In addition, emerging evidence has raised concerns regarding the efficacy of belatacept specifically in obese recipients. A pooled analysis of the BENEFIT and BENEFIT-EXT trials demonstrated that obesity was independently associated with a higher incidence of acute rejection in belatacept-treated patients [79]. This observation suggests that obesity-related factors—potentially including altered pharmacokinetics, increased clearance, or distinct immune mechanisms—may attenuate the protective effect of belatacept. Consequently, while belatacept remains a valuable option for selected obese recipients due to its favorable metabolic and renal profile, its utilization must be carefully balanced against the risk of post-transplant lymphoproliferative disorder, the need for intravenous administration, and foremost the possibility of reduced efficacy in obese patients.

## Therapeutic drug monitoring and tailoring strategies

As already discussed for the individual immunosuppressive agents, therapeutic drug monitoring (TDM) is indispensable in obese transplant recipients, but conventional trough-level monitoring may be unreliable due to obesity-related changes and disturbances. In detail, trough concentrations of tacrolimus do not consistently predict overall exposure [51, 54], and similar limitations have been reported for cyclosporine [46]. This mismatch thus raises the risk of relevant therapeutic misclassification in patients with obesity. Here, limited-sampling approaches and Bayesian AUC estimation may pave the way towards more accurate dosing strategies by providing better correlation with exposure. However, these techniques remain underutilized in current routine care [80]. Along the same lines, AUC-based monitoring correlates more closely with outcomes than fixed dosing for MMF, as shown in a multicenter trial where individualized exposure significantly improved patient outcomes [68]. Another important aspect of tailoring is the choice between weight-based and fixed-dose regimens. Weight-based dosing often leads to overexposure in obese patients, particularly for CNIs and mTOR inhibitors [49, 51]. Therefore, fixed dosing with close monitoring appears safer but is still associated with the potential risk of impaired efficacy. Although desperately needed, no consensus guidelines currently exist [45]. When striving out for concepts beyond pharmacokinetics, biomarker-based strategies may further help to refine immunosuppression intensity. Here, donor-specific antibody monitoring, immune cell functional assays (such as IFN-γ ELISPOT), and transcriptomic signatures have demonstrated additional prognostic value [81]. Therefore, integration of TDM with biomarker-based tools might represent the next level in precision transplant pharmacology [82].

## Cross-class comparison

When comparing drug classes, the individual profile of each immunosuppressive agent reveals both obesity-specific strengths and limitations. While still representing the gold standard of immunosuppression in the global population, CNIs remain highly effective in obese transplant recipients, but their use in this particular patient subgroup is complicated by pharmacokinetic variability, nephrotoxicity, and the limited reliability of trough levels; moreover, tacrolimus in particular confers a substantially increased risk of post-transplant diabetes, a complication of major concern in this population [45, 46]. Here, mTOR inhibitors provide an alternative to CNIs and allow for minimization strategies but frequently exacerbate dyslipidemia and insulin resistance, thereby worsening metabolic syndrome in obese recipients [60, 61, 83]. Mycophenolate mofetil is generally well tolerated but may exhibit exposure variability that may be even altered by obesity [50, 64]. Glucocorticoids remain the most problematic class, as their adverse metabolic effects directly overlap with the obesity phenotype, although systematic obesity-specific pharmacokinetic data are lacking [70, 71]. Of note, belatacept has emerged as an attractive therapeutic option because of its nephroprotective properties, fixed intravenous dosing, and absence of metabolic toxicity [77, 78, 84]. However, recent evidence indicates that obese recipients treated with belatacept may experience a higher incidence of acute rejection, highlighting the need for careful patient selection and close immunological monitoring [79].

Taken together, these cross-class comparisons underscore that none of the presented immunosuppressive agents provides an ideal single solution for obese graft recipients. Yet, a rational, tailored combination—balancing efficacy, nephroprotection, and metabolic safety—may offer the opportunity to optimize clinical outcomes. Framing immunosuppression within an individualized, obesity-aware therapeutic strategy may thus transform the apparent challenge into an opportunity for precision medicine in transplantation.

## Precision medicine in obese transplant recipients

Since no single immunosuppressive agent provides an ideal solution for obese transplant recipients, this obvious limitation should rather serve as the starting point for future precision approaches.

In detail, pharmacogenomic testing, most notably CYP3A5 genotyping for tacrolimus, may represent another tangible strategy for individualized dosing. Growing evidence supports its value in optimizing initial dosing, although robust outcome data in obese patient cohorts remain scarce [85–87]. In addition, advances in biomarker-based monitoring—including donor-derived cell-free DNA, microRNAs, chemokine panels, and gene expression profiling—offer further noninvasive tools for dynamic immunological risk assessment [88, 89]. The Barcelona Consensus already recommended integrating biomarkers into clinical immunosuppressive drug management, while acknowledging that most assays still remain under evaluation and are not yet ready for broad implementation [90].

Beyond pharmacogenomic profiling and pharmacokinetic monitoring, biomarker-based approaches are increasingly being explored to refine immunological risk assessment. Here, donor-derived cell-free DNA (dd-cfDNA) is currently the most advanced tool in this context and has already shown good performance for the detection of allograft injury, particularly antibody-mediated rejection, although its specificity remains limited, as elevated levels may also occur in the setting of infection or non-immune injury [89, 91].

Other platforms, including circulating microRNAs, chemokine panels, and gene expression profiling, provide complementary insights into immune activation and graft injury. Among these approaches, dd-cfDNA is currently the most clinically advanced and widely implemented one, whereas transcriptomic platforms are gaining increasing traction in selected settings. In contrast, microRNAs and chemokine-based assays remain largely investigational, with limited availability in routine clinical practice. While these approaches have shown promising diagnostic accuracy in selected settings, their clinical implementation is still limited by the lack of standardized assays and consistent validation across centers [88, 89]. Transcriptomic strategies, in particular, are increasingly incorporated into clinical algorithms, especially in heart and kidney transplantation, but their broader applicability remains under evaluation [82, 89].

Despite these advances, several barriers continue to limit the routine use of biomarker-based monitoring, including costs, limited availability outside specialized centers, and inter-variability between platforms with respect to thresholds and analytical performance [89]. Of critical relevance, prospective data demonstrating a clear impact on clinical outcomes are still scarce.

Importantly, data on biomarker performance in obese transplant recipients are largely lacking. Given that obesity is associated with chronic low-grade inflammation, altered immune cell function, and a high burden of metabolic comorbidities, biomarker readouts may be more difficult to interpret in this population [92]. This represents a relevant knowledge gap and highlights the need for further studies specifically addressing biomarker-guided immunosuppression across different metabolic phenotypes. In this context, obesity may represent a clinically relevant stress model to test and refine biomarker-driven precision immunosuppression strategies.

Of note, real-world analyses have already begun to address obese populations specifically: a propensity-matched study in kidney transplantation demonstrated that immunosuppressive protocol choice significantly influences outcomes in obese recipients, supporting the concept of tailored immunsuppressive approaches [61].

**FIGURE 3:**
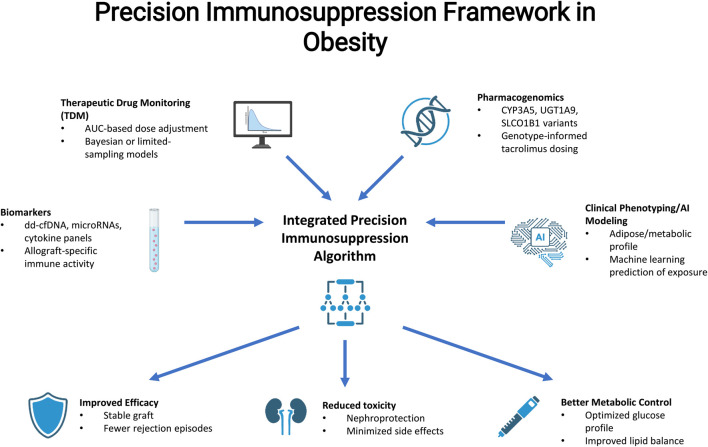
Precision immunosuppression framework for obese transplant recipients. Integrated model combining clinical phenotyping, metabolic and adipose-tissue profiling, pharmacogenomics (e.g., CYP3A5 variants), biomarker-based immunomonitoring (dd-cfDNA, microRNAs, cytokine panels), and therapeutic drug monitoring using AUC-based or Bayesian approaches. This multidimensional algorithm aims to optimize immunosuppressive exposure, reduce toxicity, stabilize graft function, and improve metabolic outcomes, outlining a precision-medicine strategy tailored to the obese transplant population.

From this perspective, obesity should not only be regarded as a potentially deleterious patient´s variable but rather as an ideal clinical scenario for the implementation of precision medicine in clinical transplantation. Here, integrating TDM, pharmacogenomics, and validated biomarkers with detailed phenotyping and computational modeling may truly enable individualized immunosuppressive regimens although further prospective validation will be crucial (see Figure 3).

Building on these concepts, a pragmatic framework for individualized immunosuppressive management in obese transplant recipients can be proposed. While high-quality prospective data are lacking, several principles emerge from available pharmacokinetic and clinical evidence. A concise overview of key drug-specific considerations is summarized in Table 1. In general, fixed or capped dosing strategies may be preferable to total body weight-based approaches for lipophilic agents such as calcineurin inhibitors, in order to reduce the risk of overexposure and toxicity. Early and repeated therapeutic drug monitoring, ideally incorporating AUC-based approaches where feasible, appears particularly important in this population, especially for agents such as tacrolimus and mycophenolate.

**TABLE 1:** Immunosuppressive drug class considerations in obese transplant recipients.

Drug class	Key considerations in obesity	Practical approach
Calcineurin inhibitors	Variable pharmacokinetics; risk of overexposure with weight-based dosing; nephrotoxicity	Prefer fixed or capped dosing; early and repeated therapeutic drug monitoring
mTOR inhibitors	Dyslipidemia, insulin resistance; impaired wound healing	Careful patient selection; close metabolic monitoring
Mycophenolate mofetil	Reduced exposure in higher body weight	Consider AUC-guided dosing where feasible
Glucocorticoids	Enhanced metabolic impairment	Early minimization depending on the immunological risk profile
Belatacept	Favorable metabolic profile; limited data in obesity	Consider in selected patients at high risk of calcineurin inhibitor toxicity

Drug class selection should be primarily considered based on the metabolic and clinical profile of the individual patient. In detail, calcineurin inhibitor minimization strategies may be considered in individuals at high risk of nephrotoxicity, whereas mTOR inhibitors require caution in patients with pre-existing dyslipidemia or impaired wound healing. Mycophenolate exposure may be reduced in obese recipients, supporting the use of exposure-guided dosing strategies. In selected cases, belatacept-based regimens may offer a metabolically favorable alternative, although data in obese populations remain limited.

Importantly, these considerations should not be interpreted as prescriptive recommendations but rather as a conceptual framework to support individualized decision-making. Integration of pharmacokinetic monitoring, emerging biomarkers, and clinical phenotyping is likely essential to optimize the balance between rejection and toxicity in this complex patient population.

In addition to immunosuppressive tailoring, adjunctive management of obesity-related metabolic risk is becoming increasingly relevant in transplant recipients. Emerging evidence suggests that glucagon-like peptide-1 receptor agonists (GLP-1RAs) and sodium–glucose cotransporter-2 inhibitors (SGLT2 inhibitors) may improve body weight, glycemic control, and cardiovascular risk profiles in selected transplant populations, particularly after kidney transplantation [93, 94]. Available studies further indicate an overall favorable safety profile, with no consistent evidence for clinically relevant interactions with standard immunosuppressive agents, although gastrointestinal intolerance with GLP-1RAs and genitourinary infections with SGLT2 inhibitors remain important considerations [93, 94].

Yet, the underlying evidence is still largely observational, and transplant-specific prospective data remain limited [93–95]. These agents should therefore be viewed as promising adjuncts within a multidisciplinary metabolic strategy rather than as established components of immunosuppressive management.

## Knowledge gaps, future directions and conclusion

Despite a growing body of evidence, important gaps of knowledge continue to limit the development of evidence-based strategies for obese transplant recipients. Most pharmacokinetic studies remain small and retrospective [47, 49, 51], and only few trials prespecify obesity as a stratification variable, thus leaving uncertainty about optimal dosing [5, 11]. Especially steroids are understudied in this vulnerable population [69, 70], and current data rarely integrate obesity-driven inflammation, pharmacokinetic variability, and clinical outcomes within the same cohorts [82]. Large datasets seldom include detailed body composition or metabolic phenotyping [43], and mechanistic insights such as histopathological correlates are only rarely linked with pharmacological and clinical data [15].

A major limitation in current practice is the reliance on body mass index as the primary measure of obesity. Additional metrics such as waist-to-hip ratio, visceral fat quantification, and CT-based skeletal muscle index provide a more refined assessment of metabolic and immunological risk and may better identify high-risk phenotypes such as sarcopenic obesity. BMI does not reflect body composition or fat distribution and therefore provides only a limited estimate of metabolic risk. In this context, visceral adiposity and ectopic fat appear to be more closely linked to metabolic and inflammatory complications than overall body weight. Similarly, sarcopenic obesity—defined by the coexistence of excess adiposity and reduced muscle mass—has been associated with frailty and poorer post-transplant outcomes [96].

Imaging-based approaches, particularly computed tomography-based assessment of skeletal muscle mass, as well as functional measures of body composition, may allow for a more accurate characterization of metabolic risk. Integrating these parameters into clinical studies and transplant registries could improve risk stratification and help refining immunosuppressive strategies according to individual metabolic risk profiles [96].

Nevertheless, these challenges outline a clear roadmap for future research. Obesity-stratified randomized controlled trials, adequately powered pharmacokinetic/pharmacodynamic studies, and systematic evaluation of AUC-based monitoring will be essential [68, 80]. Advances in model-informed precision dosing, pharmacogenomics, and biomarker-guided immunomonitoring offer unprecedented opportunities towards individualized immunosuppressive therapy [82, 97]. Integrating these tools with detailed phenotyping and computational prediction models may help to transform the current limitations into actionable strategies [97].

In summary, obesity consistently emerges as a risk factor for inferior graft outcomes, higher perioperative morbidity, and increased metabolic complications across solid organ transplantation [7, 8]. By understanding the complex interplay of obesity-induced inflammation, pharmacokinetic variability, and immunological risk [33, 38], clinicians will have to move beyond “one-size-fits-all” approaches. Here, tailoring immunosuppression through therapeutic drug monitoring, biomarker-guided adjustments, and precision dosing algorithms holds the promise of improving long-term graft survival and patient wellbeing in this high-risk population [66, 68, 82, 97]. From this perspective, obesity should not only be regarded as a clinical challenge but as an ideal context for precision medicine, where individualized strategies can turn risk into opportunity.

## References

[1] Beckmann S Drent G Ruppar T Nikolić N De Geest S . Body weight parameters are related to morbidity and mortality after liver transplantation: a systematic review and meta-analysis. Transplantation (2019) 103:2287–303. 10.1097/tp.0000000000002811 31283679

[2] Bhat M Usmani SE Azhie A Woo M . Metabolic consequences of solid organ transplantation. Endocr Rev (2021) 42:171–97. 10.1210/endrev/bnaa030 33247713

[3] Orci LA Majno PE Berney T Morel P Mentha G Toso C . The impact of wait list body mass index changes on the outcome after liver transplantation. Transpl Int (2013) 26:170–6. 10.1111/tri.12017 23199077

[4] Liese J Bottner N Büttner S Reinisch A Woeste G Wortmann M Influence of the recipient body mass index on the outcomes after kidney transplantation. Langenbecks Arch Surg (2018) 403:73–82. 10.1007/s00423-017-1584-7 28493145

[5] Yin S Wu L Huang Z Fan Y Lin T Song T . Nonlinear relationship between body mass index and clinical outcomes after kidney transplantation: a dose-response meta-analysis of 50 observational studies. Surgery (2022) 171:1396–405. 10.1016/j.surg.2021.10.024 34838329

[6] Queruel V Kabore R Guillaume A Moreau K Leffondre K Merville P Is recipient's body mass index a determinant of short term complications in early renal transplantation? Prog Urol (2020) 30:663–74. 10.1016/j.purol.2020.07.003 32826196

[7] Hill CJ Courtney AE Cardwell CR Maxwell AP Lucarelli G Veroux M Recipient obesity and outcomes after kidney transplantation: a systematic review and meta-analysis. Nephrol Dial Transpl (2015) 30:1403–11. 10.1093/ndt/gfv214 26044837

[8] Hakeem AR Cockbain AJ Raza SS Pollard SG Toogood GJ Attia MA Increased morbidity in overweight and obese liver transplant recipients: a single-center experience of 1325 patients from the United Kingdom. Liver Transpl (2013) 19:551–62. 10.1002/lt.23618 23408499

[9] Veroux M Mattone E Cavallo M Gioco R Corona D Volpicelli A Obesity and bariatric surgery in kidney transplantation: a clinical review. World J Diabetes (2021) 12:1563–75. 10.4239/wjd.v12.i9.1563 34630908 PMC8472502

[10] Hegyi PJ Soós A Hegyi P Szakács Z Hanák L Váncsa S Pre-transplant sarcopenic obesity worsens the survival after liver transplantation: a meta-analysis and a systematic review. Front Med (Lausanne) (2020) 7:599434. 10.3389/fmed.2020.599434 33392221 PMC7772841

[11] Schold JD Augustine JJ Huml AM Fatica R Nurko S Wee A Effects of body mass index on kidney transplant outcomes are significantly modified by patient characteristics. Am J Transpl (2021) 21:751–65. 10.1111/ajt.16196 32654372 PMC8905683

[12] Lafranca JA Jn IJ Betjes MG Dor FJ . Body mass index and outcome in renal transplant recipients: a systematic review and meta-analysis. BMC Med (2015) 13:111. 10.1186/s12916-015-0340-5 25963131 PMC4427990

[13] Caamiña L Pietropaolo A Basile G Dönmez MI Uleri A Territo A Does obesity really affect renal transplantation outcomes? Actas Urol Esp Engl Ed (2024) 48(48):125–33. 10.1016/j.acuroe.2023.08.007 37604402

[14] Foroutan F Friesen EL Clark KE Motaghi S Zyla R Lee Y Risk factors for 1-Year graft loss after kidney transplantation: systematic review and meta-analysis. Clin J Am Soc Nephrol (2019) 14:1642–50. 10.2215/CJN.05560519 31540931 PMC6832056

[15] Tsuboi N Okabayashi Y Shimizu A Yokoo T . The renal pathology of obesity. Kidney Int Rep (2017) 2:251–60. 10.1016/j.ekir.2017.01.007 29142961 PMC5678647

[16] Wang Y Chen X Song Y Caballero B Cheskin LJ . Association between obesity and kidney disease: a systematic review and meta-analysis. Kidney Int (2008) 73:19–33. 10.1038/sj.ki.5002586 17928825

[17] LaMattina JC Foley DP Fernandez LA Pirsch JD Musat AI D'Alessandro AM Complications associated with liver transplantation in the obese recipient. Clin Transpl (2012) 26:910–8. 10.1111/j.1399-0012.2012.01669.x 22694047 PMC3518672

[18] Nair S Verma S Thuluvath PJ . Obesity and its effect on survival in patients undergoing orthotopic liver transplantation in the United States. Hepatology (2002) 35:105–9. 10.1053/jhep.2002.30318 11786965

[19] Saab S Lalezari D Pruthi P Alper T Tong MJ . The impact of obesity on patient survival in liver transplant recipients: a meta-analysis. Liver Int (2015) 35:164–70. 10.1111/liv.12431 24313970

[20] Wei Q Wang K Yang M Chen J Shen T Song P Recipient gender and body mass index are associated with early acute rejection in donation after cardiac death liver transplantation. Clin Res Hepatol Gastroenterol (2020) 44S:100004. 10.1016/j.clirex.2020.100004 33602482

[21] Koh JH Chee D Ng CH Wijarnpreecha K Muthiah M Tan DJH Sex-based disparities in liver transplantation for hepatocellular carcinoma and the impact of the growing burden of NASH. Transpl Direct (2024) 10:e1642. 10.1097/TXD.0000000000001642 38911272 PMC11191941

[22] Rodriguez-Castro KI De Martin E Gambato M Lazzaro S Villa E Burra P . Female gender in the setting of liver transplantation. World J Transpl (2014) 4:229–42. 10.5500/wjt.v4.i4.229 25540733 PMC4274594

[23] Chouairi F Milner A Sen S Guha A Stewart J Jastreboff AM Impact of obesity on heart transplantation outcomes. J Am Heart Assoc (2021) 10:e021346. 10.1161/JAHA.121.021346 34854316 PMC9075353

[24] Kim D Kim IC Youn JC Chang WS Kim JJ Jung MH Impact of obesity on long term post heart transplantation outcomes. J Heart Lung Transpl (2025) 44:1396–404. 10.1016/j.healun.2025.04.021 40441396

[25] Gries CJ Bhadriraju S Edelman JD Goss CH Raghu G Mulligan MS . Obese patients with idiopathic pulmonary fibrosis have a higher 90-day mortality risk with bilateral lung transplantation. J Heart Lung Transpl (2015) 34:241–6. 10.1016/j.healun.2014.09.031 25447567

[26] Madill J Gutierrez C Grossman J Allard J Chan C Hutcheon M Nutritional assessment of the lung transplant patient: body mass index as a predictor of 90-day mortality following transplantation. J Heart Lung Transpl (2001) 20:288–96. 10.1016/s1053-2498(00)00315-6 11257554

[27] Kanasky WF Jr. Anton SD Rodrigue JR Perri MG Szwed T Baz MA . Impact of body weight on long-term survival after lung transplantation. Chest (2002) 121:401–6. 10.1378/chest.121.2.401 11834649

[28] Dobrzycka M Bzoma B Bieniaszewski K Debska-Slizien A Kobiela J . Pretransplant BMI significantly affects perioperative course and graft survival after kidney transplantation: a retrospective analysis. J Clin Med (2022) 11:11. 10.3390/jcm11154393 35956010 PMC9369329

[29] De Jong A Rolle A Souche FR Yengui O Verzilli D Chanques G How can I manage anaesthesia in obese patients? Anaesth Crit Care Pain Med (2020) 39:229–38. 10.1016/j.accpm.2019.12.009 32068132

[30] El-Mahrouk M Sorgnitt R Schwantzer G Karitinig R Jaradat D Mathe Z Body mass index: a key factor in surgical site infections after kidney transplantation? Int Wound J (2026) 23:e70901. 10.1111/iwj.70901 41946580 PMC13056484

[31] Hotamisligil GS Shargill NS Spiegelman BM . Adipose expression of tumor necrosis factor-alpha: direct role in obesity-linked insulin resistance. Science (1993) 259:87–91. 10.1126/science.7678183 7678183

[32] Ouchi N Parker JL Lugus JJ Walsh K . Adipokines in inflammation and metabolic disease. Nat Rev Immunol (2011) 11:85–97. 10.1038/nri2921 21252989 PMC3518031

[33] Hotamisligil GS Arner P Caro JF Atkinson RL Spiegelman BM . Increased adipose tissue expression of tumor necrosis factor-alpha in human obesity and insulin resistance. J Clin Invest (1995) 95:2409–15. 10.1172/JCI117936 7738205 PMC295872

[34] Lord GM Matarese G Howard JK Baker RJ Bloom SR Lechler RI . Leptin modulates the T-cell immune response and reverses starvation-induced immunosuppression. Nature (1998) 394:897–901. 10.1038/29795 9732873

[35] Procaccini C Jirillo E Matarese G . Leptin as an immunomodulator. Mol Aspects Med (2012) 33:35–45. 10.1016/j.mam.2011.10.012 22040697

[36] Matsuzawa Y . Adiponectin: a key player in obesity related disorders. Curr Pharm Des (2010) 16:1896–901. 10.2174/138161210791208893 20370675

[37] Mohamed-Ali V Goodrick S Rawesh A Katz DR Miles JM Yudkin JS Subcutaneous adipose tissue releases interleukin-6, but not tumor necrosis factor-alpha, *in vivo* . J Clin Endocrinol Metab (1997) 82:4196–200. 10.1210/jcem.82.12.4450 9398739

[38] Weisberg SP McCann D Desai M Rosenbaum M Leibel RL Ferrante AW Jr . Obesity is associated with macrophage accumulation in adipose tissue. J Clin Invest (2003) 112:1796–808. 10.1172/JCI19246 14679176 PMC296995

[39] Lumeng CN Bodzin JL Saltiel AR . Obesity induces a phenotypic switch in adipose tissue macrophage polarization. J Clin Invest (2007) 117:175–84. 10.1172/JCI29881 17200717 PMC1716210

[40] Kumari M Heeren J Scheja L . Regulation of immunometabolism in adipose tissue. Semin Immunopathol (2018) 40:189–202. 10.1007/s00281-017-0668-3 29209828

[41] Bai Y Sun Q . Macrophage recruitment in obese adipose tissue. Obes Rev (2015) 16:127–36. 10.1111/obr.12242 25586506 PMC4304983

[42] Fontana L Eagon JC Trujillo ME Scherer PE Klein S . Visceral fat adipokine secretion is associated with systemic inflammation in obese humans. Diabetes (2007) 56:1010–3. 10.2337/db06-1656 17287468

[43] Neeland IJ Ross R Despres JP Matsuzawa Y Yamashita S Shai I Visceral and ectopic fat, atherosclerosis, and cardiometabolic disease: a position statement. Lancet Diabetes Endocrinol (2019) 7:715–25. 10.1016/S2213-8587(19)30084-1 31301983

[44] Nicoletto BB Fonseca NK Manfro RC Gonçalves LF Leitão CB Souza GC . Effects of obesity on kidney transplantation outcomes: a systematic review and meta-analysis. Transplantation (2014) 98:167–76. 10.1097/tp.0000000000000028 24911038

[45] Naesens M Kuypers DR Sarwal M . Calcineurin inhibitor nephrotoxicity. Clin J Am Soc Nephrol (2009) 4:481–508. 10.2215/CJN.04800908 19218475

[46] Staatz CE Tett SE . Clinical pharmacokinetics and pharmacodynamics of tacrolimus in solid organ transplantation. Clin Pharmacokinet (2004) 43:623–53. 10.2165/00003088-200443100-00001 15244495

[47] Campara M Lourenco LM Melaragno JI Kaiser TE . Implications for body weight extremes in solid organ transplantation. Pharmacotherapy (2021) 41:44–58. 10.1002/phar.2493 33301647

[48] Henegar JR Bigler SA Henegar LK Tyagi SC Hall JE . Functional and structural changes in the kidney in the early stages of obesity. J Am Soc Nephrol (2001) 12:1211–7. 10.1681/ASN.V1261211 11373344

[49] Vanhove T Annaert P Kuypers DR . Clinical determinants of calcineurin inhibitor disposition: a mechanistic review. Drug Metab Rev (2016) 48:88–112. 10.3109/03602532.2016.1151037 26912097

[50] Saint-Marcoux F Vandierdonck S Premaud A Debord J Rousseau A Marquet P . Large scale analysis of routine dose adjustments of mycophenolate mofetil based on global exposure in renal transplant patients. Ther Drug Monit (2011) 33:285–94. 10.1097/FTD.0b013e31821633a6 21516060 PMC3384563

[51] Shuker N van Gelder T Hesselink DA . Intra-patient variability in tacrolimus exposure: causes, consequences for clinical management. Transpl Rev (Orlando) (2015) 29:78–84. 10.1016/j.trre.2015.01.002 25687818

[52] Andrews LM de Winter BC Tang JT Shuker N Bouamar R van Schaik RH Overweight kidney transplant recipients are at risk of being overdosed following standard bodyweight-based tacrolimus starting dose. Transpl Direct (2017) 3:e129. 10.1097/TXD.0000000000000644 28361113 PMC5367746

[53] Flechner SM Kolbeinsson ME Tam J Lum B . The impact of body weight on cyclosporine pharmacokinetics in renal transplant recipients. Transplantation (1989) 47:806–10. 10.1097/00007890-198905000-00012 2655218

[54] Borobia AM Romero I Jimenez C Gil F Ramirez E De GR Trough tacrolimus concentrations in the first week after kidney transplantation are related to acute rejection. Ther Drug Monit (2009) 31:436–42. 10.1097/FTD.0b013e3181a8f02a 19494792

[55] Ekberg H Tedesco-Silva H Demirbas A Vitko S Nashan B Gurkan A Reduced exposure to calcineurin inhibitors in renal transplantation. N Engl J Med (2007) 357:2562–75. 10.1056/NEJMoa067411 18094377

[56] Kirchner GI Meier-Wiedenbach I Manns MP . Clinical pharmacokinetics of everolimus. Clin Pharmacokinet (2004) 43:83–95. 10.2165/00003088-200443020-00002 14748618

[57] Tedesco-Silva H Saliba F Barten MJ De Simone P Potena L Gottlieb J An overview of the efficacy and safety of everolimus in adult solid organ transplant recipients. Transpl Rev (Orlando) (2022) 36:100655. 10.1016/j.trre.2021.100655 34696930

[58] Kahan BD . Sirolimus: a new agent for clinical renal transplantation. Transpl Proc (1997) 29:48–50. 10.1016/s0041-1345(96)00008-5 9123092

[59] Morath C Arns W Schwenger V Mehrabi A Fonouni H Schmidt J Sirolimus in renal transplantation. Nephrol Dial Transpl (2007) 22:viii61–viii65. 10.1093/ndt/gfm652 17890266

[60] Lebranchu Y Thierry A Toupance O Westeel PF Etienne I Thervet E Efficacy on renal function of early conversion from cyclosporine to sirolimus 3 months after renal transplantation: concept study. Am J Transpl (2009) 9:1115–23. 10.1111/j.1600-6143.2009.02615.x 19422337

[61] Serna-Higuita LM Della Penna A Guthoff M Heyne N Beer-Hammer S Nadalin S Impact of different immunosuppressive protocols on clinical outcomes in obese kidney transplant recipients: a propensity score-matched analysis. Nephrol Dial Transpl (2023) 38:2052–66. 10.1093/ndt/gfad014 36662032

[62] Kuppahally S Al-Khaldi A Weisshaar D Valantine HA Oyer P Robbins RC Wound healing complications with *de novo* sirolimus *versus* mycophenolate mofetil-based regimen in cardiac transplant recipients. Am J Transpl (2006) 6:986–92. 10.1111/j.1600-6143.2006.01282.x 16611334

[63] Hartinger JM Rysanek P Slanar O Sima M . Pharmacokinetic principles of dose adjustment of mTOR inhibitors in solid organ transplanted patients. J Clin Pharm Ther (2022) 47:1362–7. 10.1111/jcpt.13753 35934622

[64] Bullingham RE Nicholls AJ Kamm BR . Clinical pharmacokinetics of mycophenolate mofetil. Clin Pharmacokinet (1998) 34:429–55. 10.2165/00003088-199834060-00002 9646007

[65] Kaplan B Gaston RS Meier-Kriesche HU Bloom RD Shaw LM . Mycophenolic acid exposure in high- and low-weight renal transplant patients after dosing with mycophenolate mofetil in the opticept trial. Ther Drug Monit (2010) 32:224–7. 10.1097/FTD.0b013e3181d18baa 20216117

[66] van Gelder T Le Meur Y Shaw LM Oellerich M DeNofrio D Holt C Therapeutic drug monitoring of mycophenolate mofetil in transplantation. Ther Drug Monit (2006) 28:145–54. 10.1097/01.ftd.0000199358.80013.bd 16628123

[67] Shaw LM Holt DW Oellerich M Meiser B van Gelder T . Current issues in therapeutic drug monitoring of mycophenolic acid: report of a roundtable discussion. Ther Drug Monit (2001) 23:305–15. 10.1097/00007691-200108000-00001 11477311

[68] Le Meur Y Buchler M Thierry A Caillard S Villemain F Lavaud S Individualized mycophenolate mofetil dosing based on drug exposure significantly improves patient outcomes after renal transplantation. Am J Transpl (2007) 7:2496–503. 10.1111/j.1600-6143.2007.01983.x 17908276

[69] Pascual J Zamora J Galeano C Royuela A Quereda C . Steroid avoidance or withdrawal for kidney transplant recipients. Cochrane Database Syst Rev (2009) (1):CD005632. 10.1002/14651858.CD005632.pub2 19160257

[70] Vincenti F Schena FP Paraskevas S Hauser IA Walker RG Grinyo J A randomized, multicenter study of steroid avoidance, early steroid withdrawal or standard steroid therapy in kidney transplant recipients. Am J Transpl (2008) 8:307–16. 10.1111/j.1600-6143.2007.02057.x 18211506

[71] Walker BR . Glucocorticoids and cardiovascular disease. Eur J Endocrinol (2007) 157:545–59. 10.1530/EJE-07-0455 17984234

[72] Tomlinson JW Walker EA Bujalska IJ Draper N Lavery GG Cooper MS 11beta-hydroxysteroid dehydrogenase type 1: a tissue-specific regulator of glucocorticoid response. Endocr Rev (2004) 25:831–66. 10.1210/er.2003-0031 15466942

[73] Elster EA Leeser DB Morrissette C Pepek JM Quiko A Hale DA Obesity following kidney transplantation and steroid avoidance immunosuppression. Clin Transpl (2008) 22:354–9. 10.1111/j.1399-0012.2008.00792.x 18279417

[74] van Raalte DH Ouwens DM Diamant M . Novel insights into glucocorticoid-mediated diabetogenic effects: towards expansion of therapeutic options? Eur J Clin Invest (2009) 39:81–93. 10.1111/j.1365-2362.2008.02067.x 19200161

[75] Vincenti F Larsen C Durrbach A Wekerle T Nashan B Blancho G Costimulation blockade with belatacept in renal transplantation. N Engl J Med (2005) 353:770–81. 10.1056/NEJMoa050085 16120857

[76] Rostaing L Massari P Garcia VD Mancilla-Urrea E Nainan G del Carmen Rial M Switching from calcineurin inhibitor-based regimens to a belatacept-based regimen in renal transplant recipients: a randomized phase II study. Clin J Am Soc Nephrol (2011) 6:430–9. 10.2215/CJN.05840710 21051752 PMC3052236

[77] Vincenti F Rostaing L Grinyo J Rice K Steinberg S Gaite L Belatacept and long-term outcomes in kidney transplantation. N Engl J Med (2016) 374:333–43. 10.1056/NEJMoa1506027 26816011

[78] Shen J Townsend R You X Shen Y Zhan P Zhou Z Pharmacokinetics, pharmacodynamics, and immunogenicity of belatacept in adult kidney transplant recipients. Clin Drug Investig (2014) 34:117–26. 10.1007/s40261-013-0153-2 24217983 PMC3899455

[79] Lange NW King K Husain SA Salerno DM Tsapepas DS Hedvat J Obesity is associated with a higher incidence of rejection in patients on belatacept: a pooled analysis from the BENEFIT/BENEFIT-EXT clinical trials. Am J Transpl (2024) 24:1027–34. 10.1016/j.ajt.2024.02.015 38387620 PMC11930353

[80] Musuamba FT Rousseau A Bosmans JL Senessael JJ Cumps J Marquet P Limited sampling models and Bayesian estimation for mycophenolic acid area under the curve prediction in stable renal transplant patients co-medicated with ciclosporin or sirolimus. Clin Pharmacokinet (2009) 48:745–58. 10.2165/11318060-000000000-00000 19817503

[81] Lefaucheur C Loupy A Vernerey D Duong-Van-Huyen JP Suberbielle C Anglicheau D Antibody-mediated vascular rejection of kidney allografts: a population-based study. Lancet (2013) 381:313–9. 10.1016/S0140-6736(12)61265-3 23182298

[82] Naesens M Anglicheau D . Precision transplant medicine: biomarkers to the rescue. J Am Soc Nephrol (2018) 29:24–34. 10.1681/ASN.2017010004 28993504 PMC5748900

[83] Kuypers DR . Benefit-risk assessment of sirolimus in renal transplantation. Drug Saf (2005) 28:153–81. 10.2165/00002018-200528020-00006 15691225

[84] Durrbach A Pestana JM Pearson T Vincenti F Garcia VD Campistol J A phase III study of belatacept *versus* cyclosporine in kidney transplants from extended criteria donors (BENEFIT-EXT study). Am J Transpl (2010) 10:547–57. 10.1111/j.1600-6143.2010.03016.x 20415898

[85] Cheung CY Tang SCW . Personalized immunosuppression after kidney transplantation. Nephrology (Carlton) (2022) 27:475–83. 10.1111/nep.14035 35238110

[86] Turolo S Edefonti A Syren ML Montini G . Pharmacogenomics of old and new immunosuppressive drugs for precision medicine in kidney transplantation. J Clin Med (2023) 12:12. 10.3390/jcm12134454 37445489 PMC10342352

[87] Belardi R Pacifici F Baldetti M Velocci S Minieri M Pieri M Trends in precision medicine and pharmacogenetics as an adjuvant in establishing a correct immunosuppressive therapy for kidney transplant: an up-to-date historical overview. Int J Mol Sci (2025) 26:26. 10.3390/ijms26051960 40076585 PMC11900248

[88] Millan O Julian J Brunet M . miRNAs, dd-cf-DNA, and chemokines as potential noninvasive biomarkers for the assessment of clinical graft evolution and personalized immunosuppression requirement in solid organ transplantation. Ther Drug Monit (2025) 47:77–97. 10.1097/FTD.0000000000001276 39503575

[89] Loupy A Sablik M Khush K Reese PP . Advancing patient monitoring, diagnostics, and treatment strategies for transplant precision medicine. Lancet (2025) 406:389–402. 10.1016/S0140-6736(25)00195-3 40614744

[90] Brunet M Shipkova M van Gelder T Wieland E Sommerer C Budde K Barcelona consensus on biomarker-based immunosuppressive drugs management in solid organ transplantation. Ther Drug Monit (2016) 38(Suppl. 1):S1–20. 10.1097/FTD.0000000000000287 26977997

[91] Filippone EJ Farber JL . The monitoring of donor-derived cell-free DNA in kidney transplantation. Transplantation (2021) 105:509–16. 10.1097/TP.0000000000003393 32732615

[92] Zhou H Gizlenci M Xiao Y Martin F Nakamori K Zicari EM Obesity-associated inflammation and alloimmunity. Transplantation (2025) 109:588–96. 10.1097/TP.0000000000005183 39192462 PMC11868468

[93] Lee SA Verhoeff R Hullekes F Hansrivijit P de Bruin RWF Porte RJ SGLT2 inhibitors and GLP-1 receptor agonists in kidney transplantation: a systematic review and meta-analysis. Transplantation (2026) 110:e217–e28. 10.1097/TP.0000000000005496 40702593 PMC13725813

[94] Ertuglu LA Porrini E Hornum M Demiray A Afsar B Ortiz A Glucagon-like peptide-1 receptor agonists and sodium-glucose cotransporter 2 inhibitors for diabetes after solid organ transplantation. Transpl Int (2021) 34:1341–59. 10.1111/tri.13883 33880815

[95] Dotan I Rudman Y Turjeman A Akirov A Steinmetz T Calvarysky B Glucagon-like peptide 1 receptor agonists and cardiovascular outcomes in solid organ transplant recipients with diabetes mellitus. Transplantation (2024) 108:e121–e8. 10.1097/TP.0000000000004945 38361246

[96] Carey EJ Lai JC Sonnenday C Tapper EB Tandon P Duarte-Rojo A A north American expert opinion statement on sarcopenia in liver transplantation. Hepatology (2019) 70:1816–29. 10.1002/hep.30828 31220351 PMC6819202

[97] Minichmayr IK Dreesen E Centanni M Wang Z Hoffert Y Friberg LE Model-informed precision dosing: state of the art and future perspectives. Adv Drug Deliv Rev (2024) 215:115421. 10.1016/j.addr.2024.115421 39159868

